# DeStyle: A Unified Decoupled Framework for Style Transfer via Singular Value Purification and Attention-Guided Distillation

**DOI:** 10.3390/jimaging12100494

**Published:** 2026-10-08

**Authors:** Xinying Liu, Tian Xian, Xuelei Geng, Di Zhao, Wei Liu

**Affiliations:** 1 College of Intelligent Equipment, Shandong University of Science and Technology, Taian 271019, China; xinying0@aliyun.com (X.L.); xt485556164@163.com (T.X.); xueleigeng@gmail.com (X.G.); 3029307544zzik@gmail.com (D.Z.); 2 College of Computer Science and Engineering, Shandong University of Science and Technology, Qingdao 266590, China

**Keywords:** style transfer, diffusion models, singular value decomposition, attention distillation, training-free, decoupled framework

## Abstract

A major challenge in training-free image style transfer is to reproduce the artistic characteristics of a reference image while preserving the semantic identity and spatial structure of the content image. Existing methods often struggle to balance content preservation and stylistic expressiveness: aggressive feature injection may introduce semantic information from the style reference, whereas conservative strategies tend to produce only coarse color and tone transfer. To address this issue, we propose DeStyle, a unified training-free framework that progressively decouples style transfer across latent initialization, feature conditioning, and latent-manifold alignment. First, Precision Trajectory Inversion (PT-Inversion) and Dual-Priors Statistical Fusion (DPSF) establish a content-aware initial latent prior by reducing inversion errors and aligning the statistical characteristics of content and style representations. Second, a dual-branch regulation architecture is equipped with Dynamic Singular Component Filtering (DSCF), which dynamically suppresses semantic interference in stylistic features through time-aware singular value filtering. Finally, Latent-Manifold Alignment via Attention-Guided Distillation (LMA-AGD) refines the latent representation by aligning attention distributions, enabling more accurate reconstruction of fine-grained artistic textures. Extensive experiments on benchmark datasets demonstrate that DeStyle achieves superior performance in structural fidelity, content-leakage suppression, and stylistic expressiveness compared with existing training-free style transfer methods.

## 1. Introduction

In recent years, text-to-image diffusion models have demonstrated remarkable capabilities in high-fidelity image generation [1,2,3,4,5,6] and have provided new opportunities for image-to-image stylization. Image style transfer aims to render the artistic characteristics of a reference image onto a content image while preserving its semantic identity and spatial structure [7,8]. Although fine-tuning-based approaches such as Textual Inversion [9], LoRA [10], and DreamBooth [11] can effectively model specific visual concepts or styles, their computational cost and instance-specific training requirements limit their practical deployment. Consequently, increasing attention has been devoted to training-free style transfer methods that directly exploit the priors embedded in pre-trained diffusion models [12,13,14].

Despite their efficiency and flexibility, existing training-free methods face a fundamental dilemma between content leakage and stylistic mediocrity [15,16]. On the one hand, aggressive feature injection strategies can capture rich artistic characteristics but may inadvertently transfer semantic entities from the reference image, resulting in identity contamination or structural distortion [17]. On the other hand, conservative feature restriction can effectively reduce such leakage but often limits stylization to global color and tonal changes, making it difficult to reproduce high-frequency textures and fine-grained artistic patterns [18,19]. This dilemma stems from the strong entanglement of spatial structure, semantic content, and visual style within the high-dimensional feature spaces of pre-trained diffusion models [19].

To address this challenge, we propose DeStyle, a unified training-free framework that progressively decouples style transfer at three complementary stages: latent initialization, feature conditioning, and latent-manifold alignment. In the initialization stage, PT-Inversion and DPSF establish a structurally aligned and stylistically enriched latent prior before denoising. During feature conditioning, the proposed dual-branch architecture separately regulates content structure and stylistic information, while DSCF suppresses semantic interference in style features at the spectral level. Finally, LMA-AGD performs attention-guided latent optimization to refine fine-grained stylistic details that are difficult to capture through global feature injection alone. Through this progressive decoupling strategy, DeStyle aims to simultaneously preserve the structural integrity of the content image, prevent unwanted semantic transfer, and enhance the fidelity of artistic details.

The main contributions of this work are summarized as follows:

- We propose DeStyle, a unified training-free style transfer framework that progressively decouples structural, semantic, and stylistic information throughout the diffusion generation process.
- We introduce Dynamic Singular Component Filtering (DSCF), a time-aware singular-value filtering mechanism that suppresses semantically interfering components in stylistic features and thereby reduces content leakage during feature injection.
- We propose Latent-Manifold Alignment via Attention-Guided Distillation (LMA-AGD), which optimizes the latent representation through attention alignment to improve the reproduction of high-frequency artistic textures and local stylistic details.
- Extensive experiments on benchmark datasets demonstrate that DeStyle consistently outperforms existing training-free methods in quantitative and qualitative evaluations, including structural preservation, perceptual fidelity, and artistic style transfer quality.

## 2. Related Work

### 2.1. Diffusion Models and Controllable Generation

Denoising Diffusion Probabilistic Models (DDPMs) [20] and their variant, Latent Diffusion Models (LDMs) [1], have become the core architectures for high-fidelity image generation. To enable precise geometric control over pre-trained models, ControlNet [21] and T2I-Adapter [22] introduced supplementary lightweight conditioning branches, utilizing explicit spatial signals such as Canny edges, depth maps, or segmentation masks to guide the generation process [23,24]. Beyond text prompts, IP-Adapter [12] established an efficient image prompt adaptation paradigm by injecting global visual embeddings into the U-Net via a decoupled cross-attention mechanism [12]. The DeStyle framework builds upon this dual-branch architecture, employing ControlNet for rigid structural constraints while reconstructing the IP-Adapter’s injection mechanism to specialize in pure artistic stylization.

### 2.2. Training-Free Image Style Transfer

Early style transfer heavily relied on CNN-based statistical alignment (e.g., AdaIN [8]) or Generative Adversarial Networks (GANs) [25,26,27]. In the diffusion model era, training-free methods have gained prominence due to their plug-and-play adaptability. SDEdit [28] proposed adding noise to the content image followed by guided denoising, whereas inversion-based techniques like DDIM Inversion [29] and Null-Text Inversion [30] aim to reconstruct precise initial noise trajectories [30]. Plug-and-Play [13] and FateZero [31] demonstrated that intermediate diffusion features—particularly self-attention maps—contain rich local structural information [13,31]. Beyond these, several influential works in zero-shot image-to-image translation and editing, such as pix2pix-zero [14], InstructPix2Pix [32], Imagic [33], and cross-domain composites [34], have further expanded the potential of training-free manipulation by leveraging pre-trained semantic priors. However, when processing artistic images with highly complex textures, these methods frequently struggle to balance structural fidelity with stylistic expression. The PT-Inversion and DPSF modules proposed in DeStyle are designed to establish high-fidelity prior anchors in the latent space before inversion discretization errors accumulate. Additionally, recent zero-shot and training-free approaches such as StyleShot [35], StyleID [36], and DiffuseST [37] have demonstrated impressive capabilities in extracting style priors and manipulating diffusion attention mechanisms for robust stylization.

### 2.3. Attention Mechanisms and Feature Decoupling

To mitigate the content leakage issue, recent studies have shifted toward explicit decoupling within the feature space. InstantStyle [38] and its extension, InstantStyle-Plus [15], proposed injecting stylistic features exclusively at specific Transformer layers to separate layout from semantics [15,38]. CSGO [17] explored the compositional control of content and style within unified generation [17]. Furthermore, AttenST [16] and Attention Distillation [18] attempted to guide feature transfer utilizing attention maps [16,18], following the general principles of knowledge distillation [39,40]. Notably, CleanStyle [19] was the first to reveal the physical significance of singular value distributions within diffusion feature matrices [19]. Inspired by this, DeStyle introduces the DSCF mechanism, transforming static global observations into a time-aware, dynamic feature dimensionality reduction module. Combined with LMA-AGD, it facilitates progressive style alignment from the macro to the micro level, yielding significant improvements in the precise replication of complex textures.

## 3. Methodology

### 3.1. Preliminaries

#### 3.1.1. Latent Diffusion Models (LDM)

DeStyle is built upon the Latent Diffusion Model (LDM) framework [1]. Unlike pixel-space diffusion models, LDMs operate within a low-dimensional latent space Z facilitated by a pre-trained Variational Autoencoder (VAE). For a given content image $I_{c}$ and style image $I_{s}$, their latent representations are denoted as $z_{c},z_{s}\in Z$. The forward process transforms these latents into isotropic Gaussian noise $z_{T}$ through incremental noise injection. During inference, the reverse process employs a trained denoising autoencoder $\epsilon_{\theta }$ to recover the signal by minimizing:

(1) $$L=E_{z,\epsilon ∼N(0,I),t}\left[∥\epsilon -\epsilon_{\theta }\left(z_{t},t,c\right){∥}_{2}^{2}\right],$$

where *c* represents the conditioning input. DeStyle utilizes deterministic sampling trajectories, such as DDIM [29], to iteratively reconstruct stylized latents from noise.

#### 3.1.2. SVD and Feature Distribution

To achieve explicit mathematical decoupling of style and semantics, DeStyle leverages prior observations regarding singular value distributions in feature matrices [19]. For the conditional feature matrices X∈{K,V} within U-Net cross-attention layers, the Singular Value Decomposition (SVD) is defined as:

(2) $$X=U\Sigma V^{T}=\sum_{i=1}^{n}s_{i}u_{i}v_{i}^{T},$$

where Σ is a diagonal matrix containing singular values $s_{1}\geq s_{2}\geq \cdots \geq s_{n}$. Empirical evidence suggests that top singular values capture global statistics and macro-textures (style), whereas tail singular values encode fine-grained geometry and specific semantic structures (content) [19]. This energy distribution disparity allows for the suppression of content leakage via the selective manipulation of singular components.

### 3.2. Framework Overview

**Figure 1:**
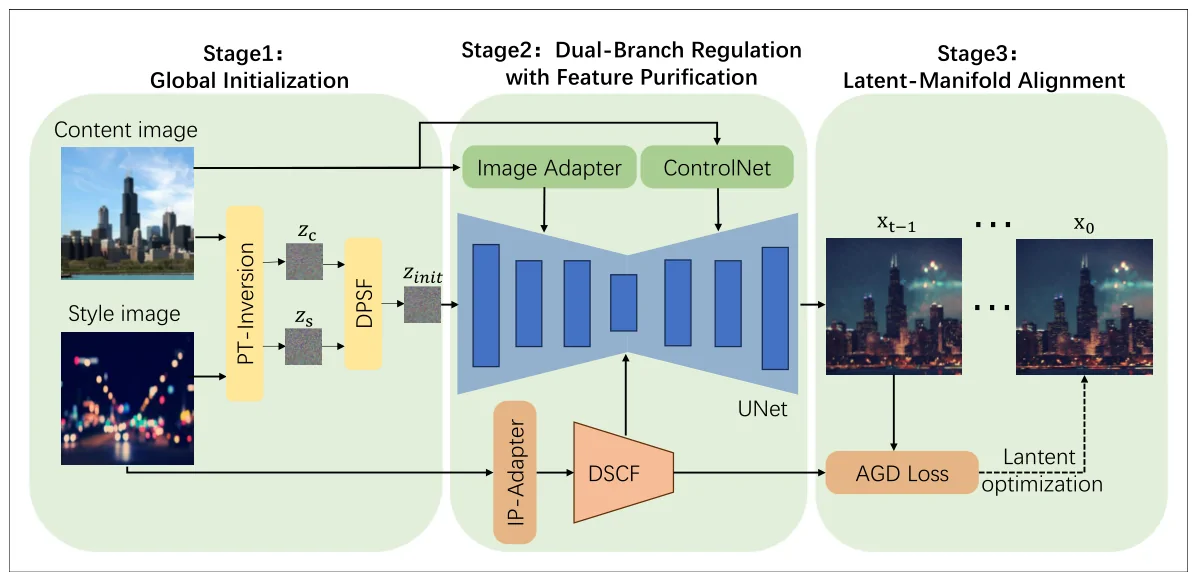
Overall architecture of DeStyle. The framework consists of three stages. **Stage 1:** Global Initialization, where PT-Inversion and DPSF construct a content-aware initial latent prior from the content and style images; **Stage 2:** Dual-Branch Regulation with Feature Purification, where the Content Branch preserves spatial structure and the Style Branch injects purified stylistic features through DSCF during denoising; and **Stage 3:** Latent-Manifold Alignment, where LMA-AGD aligns attention distributions to refine fine-grained artistic details.

The primary challenge in training-free style transfer is the entanglement of spatial structure, semantic content, and visual style within pre-trained diffusion models. To address this, we propose DeStyle, a novel and unified training-free framework that decouples the style transfer pipeline into three distinct stages (see Figure 1).

### 3.3. Global Initialization via PT-Inversion and DPSF

In the DeStyle framework, synthesis quality is significantly influenced by the initial point of the reverse denoising process on the diffusion manifold. To capture intricate artistic characteristics while ensuring structural consistency, we propose a two-stage global initialization strategy consisting of Precision Trajectory Inversion (PT-Inversion) and Dual-Priors Statistical Fusion (DPSF).

#### 3.3.1. Precision Trajectory Inversion (PT-Inversion)

Conventional inversion methods, such as the standard DDIM inversion [29], typically rely on a first-order linear approximation of the Ordinary Differential Equation (ODE) trajectory. However, under discretized sampling intervals, the discretization error inherent in single-pass forward mapping accumulates inevitably. When processing artistic images with complex textures, this cumulative bias leads to the loss of critical visual cues in the latent space, triggering structural drift or stylistic degradation during synthesis. To mitigate this, DeStyle introduces the PT-Inversion strategy, which transforms a single inversion step into an iterative Multi-resampling Correction mechanism. Within each inversion interval [t,t+1], PT-Inversion dynamically aligns the latent evolution path through iterative noise prediction refinement. This ensures that both the content image $I_{c}$ and style image $I_{s}$ are precisely projected onto the Gaussian distribution boundaries, yielding high-fidelity latent representations $z_{c}$ and $z_{s}$ that serve as robust data anchors.

#### 3.3.2. Dual-Priors Statistical Fusion (DPSF)

A primary challenge post-inversion is the seamless integration of the content image’s geometric skeleton with the global chromatic distribution of the style reference. Direct latent blending often incurs semantic ambiguity or color artifacts. Inspired by Adaptive Instance Normalization (AdaIN) [8], we developed the DPSF module to achieve deep statistical alignment between content and style features within the latent space. Instead of simple feature superposition, DPSF reconstructs the visual tone by re-modulating the statistical moments (mean μ and standard deviation σ) of the latent feature maps. We extract the spatial layout prior from $z_{c}$ and the artistic distribution prior from $z_{s}$, then derive the fused target moments using balancing factors $w_{c}$ and $w_{s}$:

(3) $$\mu_{target}=w_{s}\cdot \mu \left(z_{s}\right)+w_{c}\cdot \mu \left(z_{c}\right),$$

(4) $$\sigma_{target}=w_{s}\cdot \sigma \left(z_{s}\right)+w_{c}\sigma \left(z_{c}\right).$$

Finally, using $z_{c}$ as the structural baseline, we map its distribution into this joint probability space to construct the initialized latent $z_{init}$:

(5) $$z_{init}=\sigma_{target}⊙\left(\frac{z_{c}-\mu \left(z_{c}\right)}{\sigma (z_{c})}\right)+\mu_{target}.$$

This mechanism establishes a structurally-aligned stylistic prior in the latent space before denoising commences, effectively pre-defining the color palette while locking the geometric boundaries, thereby minimizing the risk of feature entanglement.

### 3.4. Dual-Branch Regulation via Feature Decoupling and Singular Value Purification

Following the initialization of the latent manifold, the critical challenge in the denoising inference phase is the continuous injection of high-frequency stylistic features while effectively suppressing semantic leakage from the reference image. Inspired by the layer-wise decoupling paradigm of InstantStyle [38], DeStyle introduces an augmented dual-branch regulation architecture. This framework retains the spatial flexibility of feature injection and fundamentally separates style from content at the mathematical level through Dynamic Singular Component Filtering (DSCF).

#### 3.4.1. Dual-Branch Decoupling Mechanism

To achieve precise decoupling during inference, DeStyle partitions the U-Net conditioning input stream into two parallel regulatory channels:

- **Spatial Structure Branch**: This channel enforces the geometric integrity of the source image $I_{c}$. It leverages an Image Adapter to extract global content embeddings, coupled with auxiliary conditioning networks (e.g., ControlNet [21]) that ingest explicit structural signals like Canny edges or localized image tiles. This multi-modal structural prior imposes rigid spatial constraints on the U-Net, ensuring the synthesized topology strictly aligns with the source.
- **Aesthetic Infusion Branch**: This channel adopts a feature injection paradigm, utilizing a CLIP vision encoder [41,42] to extract global features from the style image $I_{s}$, which are then mapped into the cross-attention layers via IP-Adapter [12]. While InstantStyle [38] demonstrated that layer-specific attention manipulation partially mitigates content leakage, DeStyle proposes a proactive interception strategy: applying singular value purification prior to the cross-attention mechanism, thereby blocking semantic entanglement at the root of the feature spectrum.

#### 3.4.2. Dynamic Singular Component Filtering (DSCF)

To address the persistent issue of identity leakage in style transfer, the DSCF module performs Singular Value Decomposition (SVD) on the stylistic feature matrices $X_{style}\in \left\{K,V\right\}$, formulated as:

(6) $$X_{style}=U\Sigma V^{T}=\sum_{i=1}^{n}s_{i}u_{i}v_{i}^{T},$$

where $\Sigma =\mathrm{diag}(s_{1},s_{2},\ldots ,s_{n})$ is the diagonal matrix of singular values in descending order. Empirical analysis of diffusion model feature spectra [19] indicates that the top *k* singular values primarily encode global statistical properties such as color palette and macro-textures (corresponding to style). Conversely, the tail singular values (i>k) tend to capture fine-grained topology and local semantic boundaries (corresponding to content). To achieve purified stylization, DSCF dynamically suppresses these tail components. Given that the diffusion sampling dictates global composition in early stages (high *t*) and detail refinement in later stages (low *t*), we introduce a time-aware exponential decay function f(t):

(7) $$s_{i}^{\prime }=\left\{\begin{array}{cc} s_{i}, & i\leq k\\ s_{i}\cdot \beta \cdot \exp\left(-\alpha \cdot \frac{T-t}{T}\right), & i>k \end{array}\right.,$$

where *T* represents the total denoising steps, *t* is the current step, and α,β are hyperparameters governing the filtering intensity. By reconstructing the feature matrix via an inverse SVD transformation, $X_{style}^{\prime }=U\Sigma^{\prime }V^{T}$, the pure stylistic features are subsequently injected into the U-Net. Coupled with the structural anchors from the content branch, DeStyle overcomes the limitations of existing injection paradigms, achieving a simultaneous improvement in structural fidelity and artistic expressiveness without requiring fine-tuning [19,38].

### 3.5. Latent-Manifold Alignment via Attention-Guided Distillation (LMA-AGD)

Relying solely on feature-space conditioning is often insufficient to precisely replicate intricate artistic brushstrokes and high-frequency textural details, as global embeddings lose micro-level correlations during the injection process. To bridge this visual gap, DeStyle incorporates the Latent-Manifold Alignment via Attention-Guided Distillation (LMA-AGD) mechanism [18], formulating the generation task as a latent-space optimization problem constrained by attention distributions.

#### 3.5.1. Distillation Objective

LMA-AGD enforces the current spatial attention distribution to approximate ideal artistic features during the early denoising stages—the critical window for structural and aesthetic formation. We define the activation maps of U-Net attention layers as high-dimensional manifolds capturing specific visual characteristics. Let $A_{curr}$ be the current spatial attention map output by the U-Net at timestep *t*, and $A_{target}$ be the target distribution driven by purified stylistic prompts. The LMA-AGD objective is defined as the Mean Absolute Error (L1 Loss):

(8) $$L_{LMA}=E_{layer,head}\left[∥A_{curr}-\mathrm{sg}\left(A_{target}\right){∥}_{1}\right],$$

where sg(·) is the stop-gradient operator. Minimizing $L_{LMA}$ compels the latents to form local clustering patterns akin to the style reference, effectively simulating specific micro-scale painting techniques (e.g., the fragmented brushstrokes of Impressionism or impasto textures).

#### 3.5.2. Latent-Space Backpropagation

Unlike conventional classifier guidance, LMA-AGD directly fine-tunes the latent representation $z_{t}$ during inference. For an active LMA-AGD denoising step, a forward pass computes $L_{LMA}$, followed by automatic differentiation to obtain the gradient with respect to the latent:

(9) $$g_{t}=\nabla_{z_{t}}L_{LMA}.$$

The latent is then updated via manifold optimization using an adaptive learning rate η:

(10) $$z_{t}^{*}=z_{t}-\eta \cdot \mathrm{Normalize}\left(g_{t}\right).$$

#### 3.5.3. Memory-Efficient Optimization Strategy

Executing full-gradient backpropagation on high-resolution architectures like SDXL [2] incurs prohibitive memory costs. Consequently, DeStyle implements a hybrid-precision proxy optimization strategy. The U-Net weights are frozen in half-precision (FP16) to ensure computational efficiency, while only the latent $z_{t}$ and the targeted attention processors are elevated to single-precision (FP32) to prevent numerical underflow during gradient computation. Immediately following the optimization step, the computational graph is detached, and the latent is reverted to half-precision. This mechanism enables DeStyle to capture high-frequency artistic details on consumer-grade hardware, yielding stylized outputs with highly unified brushstrokes, textures, and spatial rhythm.

## 4. Experiments and Analysis

### 4.1. Implementation Details

We adopt Stable Diffusion XL (SDXL v1.0) [2] as the generative backbone. To impose rigorous spatial constraints, ControlNet-Canny and ControlNet-Tile auxiliary models [21] are integrated into the pipeline. For feature conditioning, a CLIP-ViT-H/14 encoder [41] extracts content and style embeddings, which are subsequently injected via the IP-Adapter paradigm [12]. All experiments are conducted on a single NVIDIA 3090 (24 GB) GPU (Nvidia, Santa Clara, CA, USA) with a fixed inference resolution of 1024×1024. Regarding the generative process, the total number of DDIM inversion and inference steps is set to 50, and the generation process is initialized with a fixed random seed of 7865 to ensure strict reproducibility. For the proposed progressive decoupling modules, the core hyperparameters are configured as follows. In the LMA-AGD stage, attention distillation is activated within the first 15 steps of the overall denoising process with an optimization learning rate of η=0.02. In the DPSF module, the balancing coefficients are set to $w_{c}=0.4$ and $w_{s}=0.6$. For the DSCF module, the SVD filtering hyperparameters are set to k=1, an exponential decay factor α=3, and an initial scaling factor β=1.0 for the tail singular values.

**Dataset:** For quantitative evaluation, we construct a robust benchmark utilizing images sourced directly from the official InstantStyle-Plus repository [15] (https://github.com/instantX-research/InstantStyle-Plus, accessed on 3 September 2026). Specifically, a set of 20 content images and 105 diverse artistic style images are fully cross-paired, yielding a total of 2100 content-style evaluation pairs.

**Baselines:** We compare our proposed framework against recent state-of-the-art training-free stylization methods, including InstantStyle [38], CSGO [17], StyleShot [35], StyleID [36], and DiffuseST [37]. To ensure a strictly fair evaluation, we provide all models with the exact same source data (identical RGB content and style image pairs). To maximize their performance and respect their original architectural designs, we utilize their official open-source implementations with default optimal hyperparameters (e.g., resolution, denoising steps, initial values, and guidance settings). The variations in structural settings among the baselines reflect their inherent native mechanisms rather than disparities in the input source data.

### 4.2. Qualitative Evaluation

**Figure 2:**
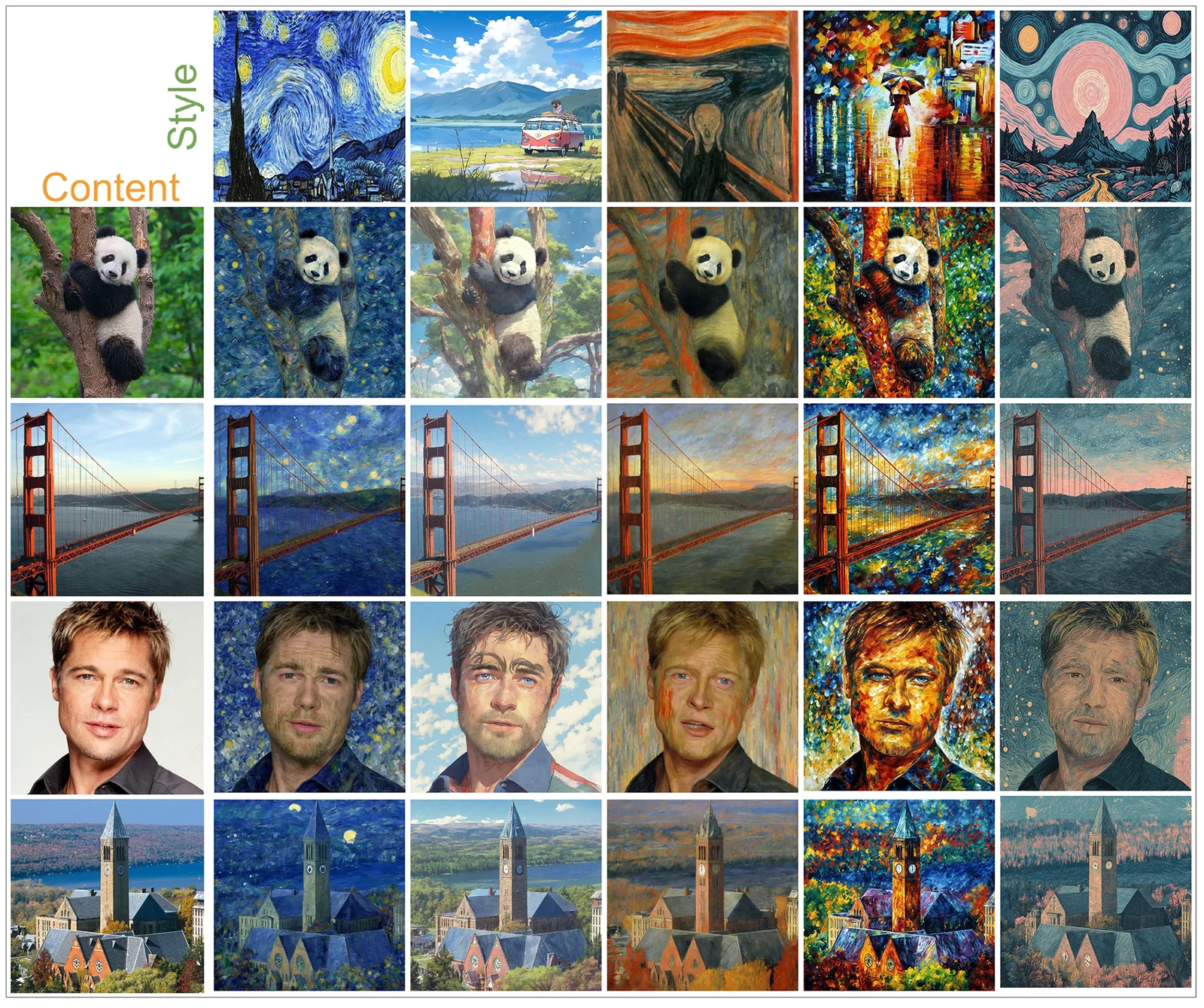
Comprehensive qualitative results of DeStyle. The matrix showcases the stylization performance across various content-style pairs. Rows represent distinct content images (e.g., natural landscapes, portraits), while columns represent diverse artistic styles (e.g., Ukiyo-e, Post-Impressionism).

To evaluate the effectiveness of DeStyle, we conduct a comprehensive qualitative comparison against the aforementioned state-of-the-art baselines. As illustrated in Figure 2, we first present the visual performance of our framework across diverse scenarios. The results demonstrate the framework’s ability to handle various content-style combinations seamlessly. As illustrated in the matrix, rows correspond to distinct content sources, while columns showcase diverse artistic genres. DeStyle consistently achieves high-quality stylization, precisely capturing stylistic nuances while maintaining the underlying geometric structure of the content images.

**Figure 3:**
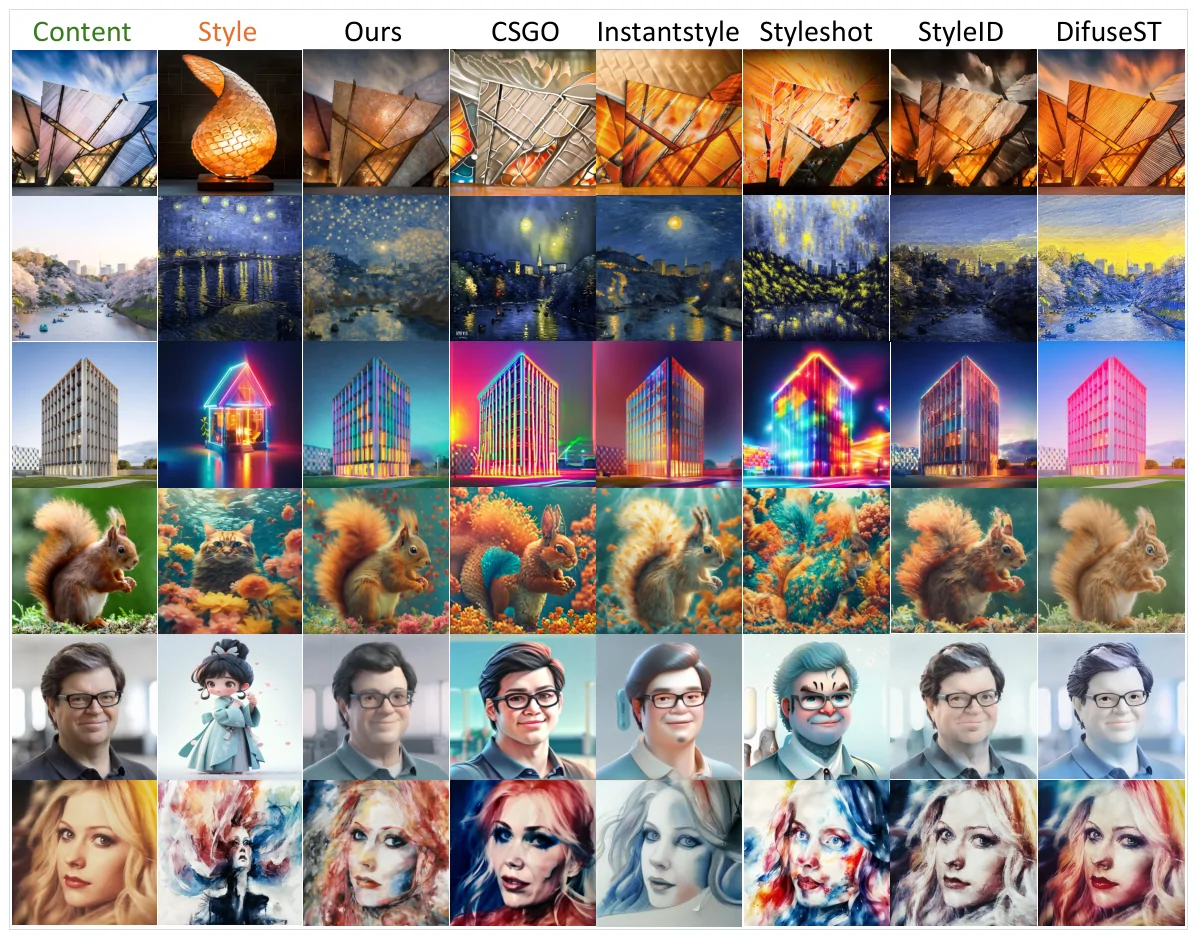
Qualitative visual comparison between DeStyle and state-of-the-art baseline models.

Furthermore, Figure 3 provides a multi-dimensional visual comparison between DeStyle and mainstream baseline models. Qualitative analysis indicates that DeStyle exhibits significant advantages across three core dimensions: First, regarding structural fidelity, DeStyle demonstrates exceptional robustness in maintaining the underlying physical structures and fine-grained semantics of the source content. For instance, in the architectural transformations (rows 1 and 3), our method accurately preserves sharp edges and spatial perspectives. Similarly, in the human portraits (rows 5 and 6), it successfully retains facial identities without distortion. Conversely, baseline methods such as CSGO [17] and StyleShot [35] frequently suffer from severe structural collapse and local artifacts, such as the facial distortion observed in row 5. This robust preservation visually validates the effectiveness of our PT-Inversion trajectory reconstruction and explicit spatial constraints. Second, in terms of disentanglement and the mitigation of content leakage, our framework effectively decouples semantic content from the reference image to extract purely stylistic representations. This capability is particularly evident in the fourth row (squirrel), where DeStyle seamlessly transfers the warm palette and painterly brushstrokes without copying unwanted semantic objects. In contrast, methods like CSGO [17] exhibit severe content leakage, erroneously transferring distinct blue pixel patches from the reference background into the synthesized output. This confirms the robust performance of the DSCF module in dynamically filtering semantic tail components at the spectral level. Finally, regarding high-fidelity style injection and visual consistency, DeStyle achieves remarkable style generalization and rendering depth while strictly preserving content. Whether applying the warm illumination in row 1, the complex starry brushstrokes in row 2, or the smooth 3D rendering in row 5, DeStyle ensures natural and comprehensive stylistic integration. In contrast, StyleID [36] and DiffuseST [37] demonstrate insufficient stylization, relying on superficial color blending as seen in rows 3 and 6, while InstantStyle [38] tends to produce muddy results with a noticeable loss of detail. The precise rendering of these nuanced artistic features highlights the crucial role of the LMA-AGD mechanism [18] in capturing micro-scale textural manifolds.

### 4.3. Quantitative Evaluation

**Metric Comparison**: To objectively assess synthesis quality, we employ LPIPS [43] to evaluate structural preservation, FID [44] to measure generative realism, and ArtFID [45] to comprehensively gauge style transfer fidelity.

Quantitative comparisons with current mainstream style transfer methods are presented in Table 1. As shown, our method significantly outperforms all baseline approaches on the ArtFID metric (31.465), which consistently aligns with human visual preferences for stylization quality. Furthermore, the proposed method achieves the lowest FID score (19.118), indicating that the generated images possess high visual fidelity and share a highly similar distribution with the target artistic styles. Regarding content preservation, DeStyle secures the lowest LPIPS score (0.564). This demonstrates that even when executing aggressive and deep style injections, our method effectively maintains the perceptual similarity of facial features and core geometric structures.

**Figure 4:**
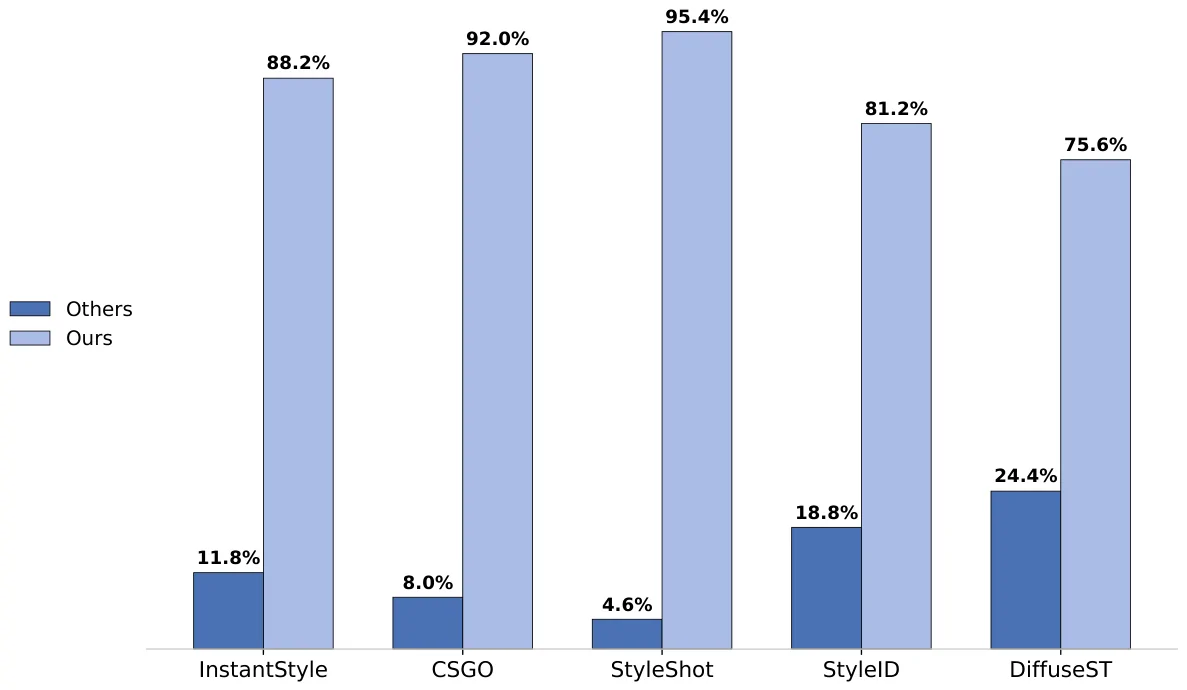
The user study compares our method with five competing methods. Users selected the preferred result between our method and each competing method. The figure shows the percentage of participants who selected each method.

**User Study**: To further evaluate the perceptual quality of the generated stylized images, we conducted a user study with 50 participants. Each participant was presented with 50 comparison questions, including 10 comparisons between our method and each of the five selected competitors. During the study, all images were displayed in a randomized and anonymous order, without revealing the corresponding generation methods, to avoid potential bias. Participants were asked to select the image with better overall visual quality based on their subjective preference. The evaluation considered the overall performance of style transfer, including style fidelity, content structure preservation, and visual coherence. In total, we collected 2500 preference votes from all participants. The voting results between our method and different competing methods are summarized in Figure 4. As shown in the figure, our method consistently achieves higher preference rates against all competing methods, demonstrating its superiority in generating visually appealing stylized images while maintaining the original content structure.

### 4.4. Comparison with Fine-Tuning Methods

**Figure 5:**
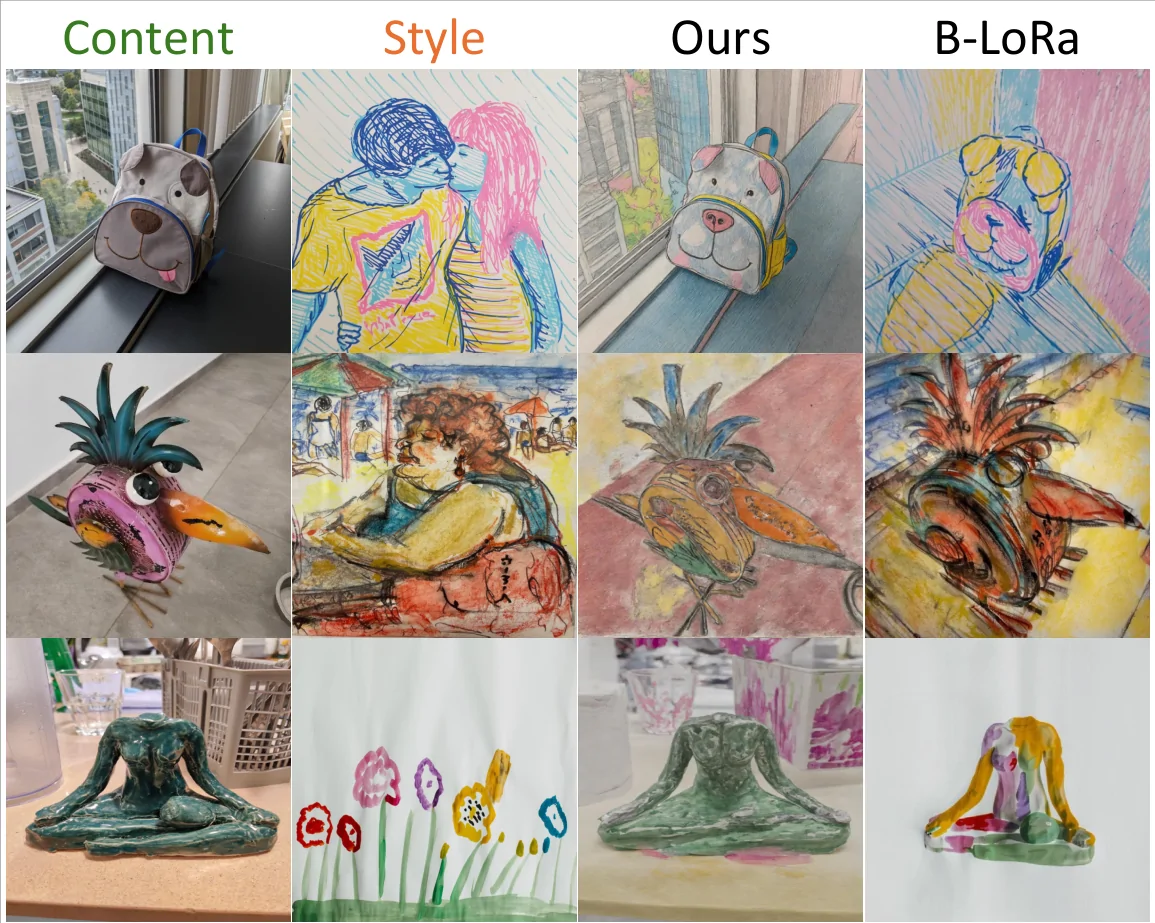
Visual comparison between our training-free DeStyle and the fine-tuning-based B-LoRA.

To rigorously position DeStyle within the broader landscape of diffusion-based stylization, we conduct a comparative evaluation against B-LoRA [46], a state-of-the-art single-image fine-tuning method. As depicted in Figure 5, B-LoRA, leveraging its training-based optimization, exhibits a strong capacity to capture and replicate the specific brushstrokes and texture distributions of the style reference. However, this aggressive textural fitting often comes at the expense of structural fidelity to the content image. For example, in the first row, B-LoRA loses fine-grained structural details such as the backpack’s handle and eyes, and even hallucinates an unnatural reflection artifact at the bottom. Similarly, the structural integrity of the bird’s tail in the second row is entirely lost. In contrast, DeStyle perfectly preserves these intricate structural semantics without introducing hallucinated elements.Furthermore, DeStyle achieves superior global stylistic harmony and scene context preservation. As observed in the third row, B-LoRA confines the stylistic transformation exclusively to the foreground sculpture and aggressively erases the complex background (e.g., the cluttered desk) into a pure white blank space. This loss of context results in a severe visual mismatch between the subject and its environment. DeStyle, conversely, applies a cohesive stylistic transformation across both the foreground subject and the contextual background, yielding a more unified and aesthetically natural artistic composition. This paradigm difference reveals a clear trade-off between offline preparation overhead and runtime efficiency. Evaluated on our hardware equipment, B-LoRA requires approximately 38 min of GPU training time to fit a single style reference, followed by a fast forward-pass inference time of about 1 min per image. In stark contrast, DeStyle operates entirely training-free, incurring zero offline preparation cost. Although its optimization-based latent guidance results in a slightly longer per-image inference latency (approximately 2.5 min), the overall “time-to-first-result” is drastically reduced from nearly 40 min to just 2.5 min.

Consequently, DeStyle and fine-tuning paradigms occupy complementary niches in practical applications. Methods like B-LoRA remain highly preferred in industrial batch-production scenarios where a fixed artistic style is applied to hundreds of visual assets, allowing the initial 38 min training cost to be effectively amortized over fast inferences. Conversely, DeStyle is distinctly advantageous in open-world, interactive, and rapid-prototyping environments where stylistic exemplars change dynamically. In such zero-shot regimes where single-use stylization is required, DeStyle completely eliminates the prohibitive training overhead, offering an optimal balance between immediate deployment and high-fidelity artistic expression.

### 4.5. Ablation Study

**Figure 6:**
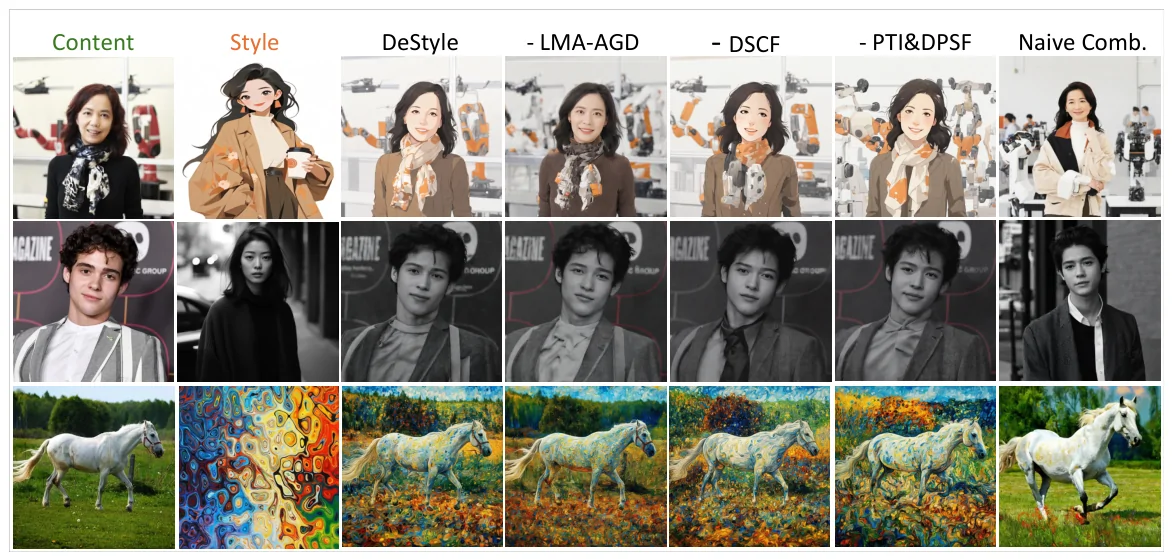
Visual ablation study of DeStyle’s core modules. We compare the full model against variants with individual components removed (w/o LMA-AGD, w/o DSCF, w/o PT-Inversion & DPSF), alongside a temporally unconstrained “Naive Combination” baseline.

To validate the individual contributions and synergistic effects of the core modules in the DeStyle framework (Global Initialization, DSCF feature purification, and LMA-AGD), we conducted a comprehensive ablation study. Visual comparisons between the full model and three ablated variants are presented in Figure 6. Additionally, the quantitative results of the ablation study, evaluated by LPIPS, FID, and ArtFID, are summarized in Table 2.

#### 4.5.1. Removing Global Initialization (w/o PT-Inversion & DPSF)

In this variant, we removed the PT-Inversion and DPSF modules, reverting to standard DDIM inversion [29] and directly utilizing the style latent $z_{s}$ as the denoising starting point. As observed in the severely degraded background robotic arms (first row) and the fragmented anatomy of the horse (third row) in Figure 6, the absence of PT-Inversion’s multi-resampling leads to severe accumulation of discretization errors. Furthermore, the lack of channel-wise alignment via DPSF deprives the latent space of the content’s geometric priors from the outset. Consequently, while this variant captures global colors, it suffers from irreversible structural distortion and localized artifacts, confirming the indispensability of global initialization for compositional anchoring. This visual observation is consistent with the quantitative metrics in Table 2. The removal of the global initialization module leads to a substantial increase in the LPIPS score, indicating a degradation in structural fidelity, which concurrently affects the overall artistic distribution (FID and ArtFID).

#### 4.5.2. Removing Feature Purification (w/o DSCF)

To evaluate the role of the DSCF module in preventing content leakage, we disabled the SVD filtering mechanism in the style branch, allowing raw CLIP visual features to directly enter the cross-attention layers. As visually evidenced in the second row of Figure 6, unfiltered feature injection triggers significant semantic leakage—specific semantic entities and identity traits from the style reference (such as the woman’s hairstyle, feminine facial characteristics, and dark coat) are erroneously mapped and superimposed onto the male subject, severely compromising content preservation. This empirical evidence supports our theoretical premise: truncating tail singular values is a mathematically necessary operation to thoroughly disentangle style from semantics and achieve purified conditioning. Quantitatively, this semantic leakage disrupts the target artistic distribution. As shown in Table 2, this is reflected by the degradation in both FID and ArtFID scores compared to the full model, alongside a compromised LPIPS due to structural occlusion.

#### 4.5.3. Removing Attention Distillation (w/o LMA-AGD)

In this variant, we disabled latent-manifold alignment, relying solely on the feature-level injection of the dual-branch architecture. Although the images preserve spatial structure without semantic leakage, the stylization is restricted to macroscopic color and tone transfer. As clearly demonstrated in the third row of Figure 6, the generated outputs entirely lack the high-frequency textural details critical for artistic expressiveness, such as the distinctive swirling impasto brushstrokes of the style reference. The inclusion of the LMA-AGD mechanism rectifies this deficiency, proving that accurate micro-texture rendering relies fundamentally on direct gradient optimization within the latent space. As shown in Table 2, the absence of this distillation mechanism negatively impacts the overall stylization fidelity. While macro-structures are preserved, the lack of high-frequency textural rendering results in a suboptimal ArtFID score compared to the fully equipped DeStyle framework.

#### 4.5.4. Comparison with Naive Combination Baseline

As a further extension of the ablation study, we first articulate the essential algorithmic differences between our core modules and traditional analogous methods. Unlike traditional AdaIN that continuously normalizes features across all timesteps, our DPSF, coupled with PT-Inversion, acts strictly as a one-time global anchor at the initial state (t=T). This anchors the macroscopic layout without continuously disrupting the model’s native generative manifold. Furthermore, in contrast to the rigid, static singular value truncation seen in methods like CleanStyle, our DSCF introduces a dynamic, time-aware exponential decay mechanism (α,β). This allows for full stylistic injection during early stages while aggressively suppressing semantic leakage later. Finally, rather than applying continuous proxy gradient optimization across all timesteps—which often overwrites content semantics—our LMA-AGD restricts attention distillation exclusively to the early structural window to avoid latent space over-fragmentation.

Based on these theoretical distinctions, we construct a “Naive Combination” baseline to provide a direct empirical comparison. This baseline removes the specific algorithmic redesigns and temporal isolation mechanisms proposed in our study, directly concatenating the standard analogous methods: it utilizes standard DDIM inversion [29] without our global anchor, applies static SVD feature truncation without time-decay, and performs continuous, unconstrained attention distillation across the entire 50-step generation process.

As visually demonstrated in the rightmost column of Figure 6, this naive combination leads to structural collapse, and completely fails to capture the vibrant abstract brushstrokes of the style reference. This dual failure is theoretically rooted in severe gradient conflicts and a fundamental violation of the intrinsic generative dynamics of diffusion models. When standard intervention techniques are naively stacked, their objective gradients entangle and conflict throughout the denoising process. For instance, continuous forced texture distillation overrides the model’s native semantic refinement updates, while the absence of an initial anchor leaves the latent space misaligned from the outset. DeStyle achieves superior generation quality precisely because it employs a strict temporal decoupling mechanism to eliminate such mutual interference. By meticulously isolating spatial anchoring, feature purification, and texture distillation into their respective optimal temporal windows, DeStyle transforms potentially competing modules into a highly synergistic framework. Consequently, it completely aligns with the native temporal manifold of the diffusion model, achieving an optimal balance between content fidelity and stylistic expressiveness.

### 4.6. Hyperparameter Sensitivity Analysis

To justify our empirical selection of the core hyperparameters in the DSCF module, we detail the theoretical rationale for the initial scaling factor β and present a visual sensitivity analysis for the top-*k* singular value threshold and the exponential decay factor α. Specifically, β acts as the initial amplitude of the tail singular values at the onset of the denoising process. Setting β=1.0 guarantees an unbiased style initialization. During the very early stages of diffusion—where the global layout and macroscopic color distributions are fundamentally formed—the complete stylistic feature space is injected without artificial suppression, ensuring maximum stylistic vibrancy. As the denoising trajectory progresses into the structure-formation phase, the time-aware exponential decay naturally takes over. A sub-optimal β<1 would prematurely weaken the stylistic impact at the crucial initialization stage, leading to dull colors, whereas β>1 would unnaturally distort the latent manifold.

**Figure 7:**
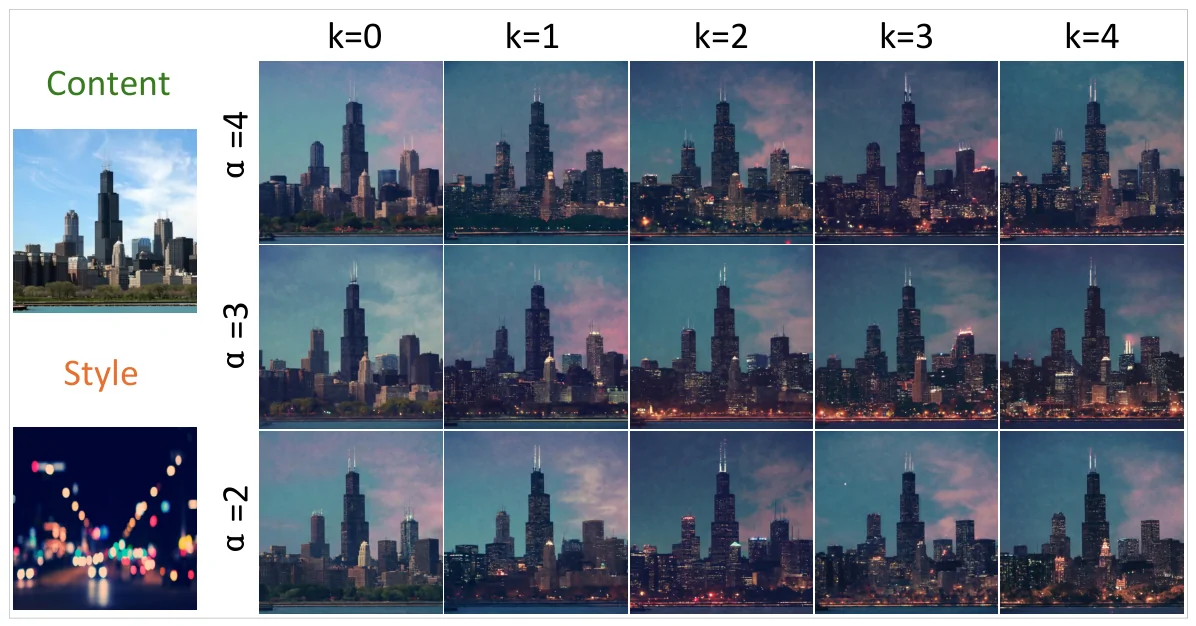
Visual sensitivity analysis of hyperparameters *k* and α.

Building upon this baseline initialization, we evaluate the impact of *k* and α, as visually illustrated in Figure 7. The parameter *k* determines how many leading singular values bypass the time-aware attenuation. As shown across the horizontal axis of Figure 7, setting k=0 results in an under-stylized image where the vibrant night-time atmosphere is insufficiently transferred. Conversely, when k≥2, specific semantic structures from the style image—such as the distinct bokeh light circles—erroneously leak into the content image, overlaying unnatural bright artifacts onto the sky and buildings. Setting k=1 successfully prevents this structural leakage while perfectly transferring the global stylistic atmosphere, such as the rich purplish color palette.

Furthermore, the parameter α controls the decay rate of the tail singular values. We evaluate the hyperparameter settings corresponding to α∈{2,3,4}. As observed in the vertical comparisons in Figure 7, a smaller value (α=2) results in a slower decay, which fails to adequately suppress the style’s spatial structures, leading to subtle structural leakage and a hazy, less clean composition in the sky. A larger value (α=4) suppresses the stylistic features too aggressively, resulting in a darker, slightly washed-out stylization where the vibrant artistic lighting effect is diminished. Consequently, setting α=3 yields the optimal balance, ensuring a smooth suppression curve that preserves content fidelity while maximizing stylistic richness.

### 4.7. Computational Efficiency and Quality-Speed Trade-Off

To comprehensively evaluate the operational efficiency and scalability of our framework, we detail the quantitative overhead across varying resolutions in Table 3. Evaluated on the same hardware environment as our previous experiments, our default high-resolution setting of 1024×1024 requires approximately 151 s to generate a single image, with a peak memory footprint of 20.0 GB. Supported by hybrid-precision (FP16) optimization, this memory requirement is successfully maintained well within the capacity of standard 24 GB consumer-grade GPUs. Furthermore, as shown in Table 3, the computational overhead exhibits a proportional scaling behavior. When adapting the framework to lower resolutions such as 768×768 and 512×512, the inference time decreases to 71 s and 66 s, respectively, accompanied by a corresponding reduction in peak VRAM. This demonstrates that our framework not only supports robust high-resolution generation but is also highly flexible in accommodating varying hardware constraints.

This difference in computational demands fundamentally reflects the inherent quality-speed trade-off in diffusion-based stylization. Within the spectrum of stylization methodologies, DeStyle occupies a strategic middle ground. On one hand, compared to purely feed-forward methods that require only a single-pass forward generation, DeStyle compromises on raw inference speed. However, lacking deep spatial optimization, pure feed-forward methods often struggle to simultaneously balance complex texture imprinting and strict structural preservation in zero-shot scenarios; DeStyle overcomes this technical bottleneck by trading a degree of inference efficiency for ultimate artistic fidelity via latent-space gradient optimization (LMA-AGD). On the other hand, compared to fine-tuning paradigms (e.g., B-LoRA), DeStyle completely circumvents the nearly 40 min offline training overhead, drastically compressing the overall “time-to-first-result” to merely a minute or two, achieving true plug-and-play capability. Consequently, our framework is not positioned for highly latency-sensitive real-time interactive scenarios but is instead dedicated to high-quality offline image creation, asynchronous asset generation, and intricate artistic prototyping; in these applications, uncompromising visual quality and zero training data preparation are prioritized over absolute generation speed.

## 5. Conclusions

In this paper, we propose DeStyle, a unified training-free image style transfer framework designed to mitigate the trade-off between stylistic expressiveness and structural fidelity in pre-trained diffusion models, with a specific focus on suppressing content leakage. Distinct from direct feature injection methods, DeStyle decouples the stylization pipeline into three progressive dimensions: macro-statistical anchoring, meso-semantic blocking, and micro-texture rendering. Specifically, through Precision Trajectory Inversion (PT-Inversion) and Dual-Priors Statistical Fusion (DPSF), the model constructs a structurally and stylistically aligned initial prior in the latent space before denoising commences, effectively mitigating feature loss caused by the accumulation of discretization errors. During the conditioning phase, the dual-branch regulation architecture integrates the Dynamic Singular Component Filtering (DSCF) module, which applies a time-aware function to dynamically truncate the tail singular values of stylistic features, achieving the separation of semantic components from artistic attributes at a mathematical level. Furthermore, to address the limitations of global feature injection in representing high-frequency artistic details, the Latent-Manifold Alignment via Attention-Guided Distillation (LMA-AGD) mechanism performs direct target texture fitting in the latent space through mixed-precision gradient optimization, enabling the accurate replication of micro-scale brushstrokes. Experiments demonstrate that DeStyle outperforms existing training-free SOTA methods across quantitative metrics—including LPIPS, FID, and ArtFID—as well as in qualitative visual assessments. This study validates the feasibility of achieving high-fidelity style transfer through thorough feature decoupling and latent-space optimization. Future work will explore extending this feature purification and distillation paradigm to video style transfer tasks, alongside further optimizing the inference overhead of gradient computations to support real-time interactive applications.

**Limitations:** Despite the significant advancements introduced by DeStyle, extensive stress testing reveals the limitations of this paradigm under extreme boundary conditions. First, regarding feature decoupling, when the style reference contains highly structured textural elements (e.g., strong geometric color blocks) or shares highly overlapping domain semantics with the content image, the texture and structure of the style become deeply entangled within the latent space. Consequently, relying solely on global linear singular value truncation struggles to find a perfect hyperplane between completely blocking semantic leakage and maintaining artistic vibrancy. Second, in terms of structural preservation, while LMA-AGD accurately anchors global layouts, it risks homogenizing micro-level local topologies (e.g., ultra-fine attributes or dense high-frequency patterns). This is fundamentally bottlenecked by the inherently low spatial resolution of the deep self-attention layers in the U-Net, where proxy gradient updates tend to smooth out dense, fine-grained semantics.

**Future Work:** To address these inherent limitations, our future work will focus on two primary directions. To overcome the entanglement of complex textures, we plan to explore advanced feature decoupling mechanisms beyond global linear transformations. By investigating localized or non-linear separation strategies, we aim to more accurately handle highly structured stylistic representations. Furthermore, to mitigate the loss of micro-level topologies, we will focus on researching multi-scale guidance frameworks. By incorporating higher-dimensional spatial constraints during the optimization process, we aim to robustly preserve fine-grained semantics, ultimately enhancing the robustness of the training-free stylization paradigm under stricter high-fidelity standards.

## Figures and Tables

**Table 1:** Quantitative comparison of DeStyle and other SOTA methods.

Method	LPIPS ↓	FID ↓	ArtFID ↓
InstantStyle [38]	0.673	21.985	38.454
CSGO [17]	0.714	22.007	39.434
StyleShot [35]	0.706	22.187	39.557
StyleID [36]	0.613	20.411	34.536
DiffuseST [37]	0.697	21.251	37.760
**DeStyle (Ours)**	**0.564**	**19.118**	**31.465**

**Table 2:** Quantitative results of the ablation study.

Method	LPIPS ↓	FID ↓	ArtFID ↓
w/o PT-Inversion & DPSF	0.685	22.104	38.930
w/o DSCF	0.642	22.450	38.505
w/o LMA-AGD	0.582	21.240	35.184
**DeStyle (Full)**	**0.564**	**19.118**	**31.465**

**Table 3:** Scalability analysis of DeStyle. The per-image inference time and peak memory consumption (VRAM) are evaluated across varying generation resolutions.

Resolution	Inference Time (s) ↓	Peak VRAM (GB) ↓
512×512	66	17.8
768×768	71	18.5
1024×1024	151	20.0

## Data Availability

The datasets presented in this article are not readily available due to technical limitations. Requests to access the datasets should be directed to the corresponding author.
